# Analysis of HIV-1 recent infections and hotspot areas in a bordering area in Yunnan Province, China

**DOI:** 10.1186/s12889-025-21877-3

**Published:** 2025-03-24

**Authors:** Xiao Zhang, Lijuan Dong, Tao Zhou, Huichao Chen, Yu Han, Xiaomei Jin, Jie Dai, Min Yang, Zhijun Zeng, Pengyan Sun, Yanling Ma, Yuhua Shi, Min Chen, Manhong Jia

**Affiliations:** 1 Yunnan Provincial Key Laboratory of Public Health and Biosafety & School of Public Health, Kunming Medical University, Kunming, Yunnan China; 2 Yunnan Provincial Key Laboratory of Public Health and Biosafety & Institute for AIDS/STD Control and Prevention, Yunnan Center for Disease Control and Prevention, Kunming, Yunnan China; 3 Division for AIDS/STD Control and Prevention, Honghe Center for Disease Control and Prevention, Honghe, Yunnan China; 4 Yunnan Provincial Key Laboratory of Public Health and Biosafety & Health Laboratory Center, Yunnan Center for Disease Control and Prevention, Kunming, Yunnan China

**Keywords:** HIV-1, Recent infection, Spatial analysis, Yunnan

## Abstract

**Background:**

Honghe Prefecture, located on the China-Vietnam border, has long suffered from HIV-1. To accurately assess the HIV-1 prevalence situation and promote precise prevention and treatment of AIDS, a recent infection surveillance was conducted to explore the at-risk subpopulations and hotspot areas of HIV-1 transmission in Honghe Prefecture.

**Methods:**

Combined with the recent infection testing algorithm, HIV-1 recency assay was used to differentiate recent HIV-1 infections among newly reported HIV-1 cases in Honghe Prefecture from 2021 to 2022. Factors associated with recent HIV-1 infection were analyzed by logistic regression. The hotspot areas of recent infections were analyzed by spatial scanning statistics.

**Results:**

Of the 2698 HIV-1-infected individuals enrolled in this study (no HIV-2 cases reported), 297 HIV-1 cases were classified as recent HIV-1 infection, and the proportion of recent infection was 11.0%. Females (AOR = 2.61, 95% *CI*: 1.98–3.43), young people (15–34 years old) (AOR = 1.71, 95% *CI*: 1.21–2.43), highly educated people (AOR = 2.04, 95% *CI*: 1.24–3.37), men who have sex with men (MSM) (AOR = 3.08, 95% *CI*: 1.74–5.46), and spouses/regular sexual partners of HIV-1-positive individuals (AOR = 1.96, 95% *CI*: 1.15–3.33) were more likely to be detected as recent infections. Among the subpopulations by sex, age and transmission route, heterosexually exposed women aged 15–34 years (OR = 1.85, 95% CI: 1.07–3.19) and 35–49 years (OR = 1.65, 95% CI: 1.05–2.58) and MSM aged 15–34 years (OR = 5.11, 95% CI: 2.73–9.59) had a higher proportion of recent infections. Among the subpopulations by mode of exposure and sex, men infected through homosexual contact (OR = 5.59, 95% *CI*: 3.30–9.47) and women infected through non-marital non-commercial heterosexual contact (OR = 2.10, 95% *CI*: 1.50–2.95) and positive spousal exposure (OR = 3.03, 95% *CI*: 1.96–4.69) had a higher proportion of recent infections. With regard to detection methods, women detected by provider-initiated HIV testing counseling (PITC) (OR = 2.03, 95% *CI*: 1.50–2.76) and spouse/sexual partner testing (OR = 4.10, 95% *CI*: 2.19–7.67) had a higher proportion of recent infections. A statistically significant spatial cluster of recent infections was found in one county, Yuanyang.

**Conclusions:**

This study investigated the use of HIV-1 recency testing in combination with HIV-1 case report surveillance. Correlation factor analysis revealed the presence of distinct risk subpopulations. The spatial distribution of recent infections showed differences. These findings were important for assessing the transmission risk and developing the targeted measures for interventions.

**Clinical trial number:**

Not applicable.

**Supplementary Information:**

The online version contains supplementary material available at 10.1186/s12889-025-21877-3.

## Introduction

Despite global efforts, it is still challenge to achieve the target of reducing new HIV infections to 335,000 by 2030 [1, 2]. HIV-infected individuals are particularly infectious during the early stages of infection (acute and recent HIV infection). Research indicates that half of all HIV transmissions occur during these early stages, highlighting the importance of identifying recent infections [3, 4]. Timely detection of recent HIV infection is crucial for two main reasons: (i) to provide behavioral interventions and antiviral therapy, thereby reducing the risk of secondary transmission; and (ii) to understand the characteristics of those recently infected, guiding targeted interventions based on behavioral and biological risks [5].

HIV recency assays utilize biomarkers to determine if an individual’s HIV-1 infection is recent (within one year of infection, with a confirmed negative test result from the previous year) or longstanding [6]. Traditionally, these assays have been used to estimate incidence rates [7]. However, recent efforts have focused on integrating recency assays into case surveillance systems and routine HIV testing services. This integration aims to measure additional indicators beyond incidence and to develop strategies for identifying hotspot populations and areas with high HIV transmission rates or emerging cases [6, 8, 9]. This approach enhances our understanding of HIV infection dynamics and supports effective control measures. Research on recent HIV infection is therefore vital for risk assessment, targeted interventions, and precision prevention and control, although further refinement is needed for non-incidence surveillance applications [9, 10].

Yunnan Province is located in the southwestern border region of China, bordering Myanmar, Laos and Vietnam, and is one of the provinces hardest hit by HIV-1 in China [11]. By the end of 2021, Yunnan Province had reported a cumulative total of 174,510 HIV/AIDS cases, with 124,372 people still alive [12]. Honghe Prefecture is located in the southeast of Yunnan, bordering Vietnam. Since 1995, when the first HIV-1 case was detected among the intravenous drug users (IDUs) in Honghe Prefecture, the number of HIV-1 infections in the region had increased [13]. A cross-sectional study of recent HIV-1 infections conducted in Yunnan Province in the past revealed a spatial clustering of recent infections in Honghe Prefecture and its neighboring Wenshan Prefecture, suggesting a higher incidence of new infections in the region [14]. However, studies focusing specifically on Honghe Prefecture have been limited primarily to examining HIV-1 prevalence rates without in-depth analysis of the characteristics and associated factors of recent infections within the prefecture. There is therefore a need to explore more comprehensive epidemic surveillance and to capture the epidemic characteristics and transmission risks of AIDS in a timely manner. Among these, recent HIV-1 infections are an important indicator for assessing AIDS trends [15]. In order to further identify high-risk populations and areas in the region, and to explore the risk of HIV-1 transmission, this study conducted a survey of newly infected persons among newly reported HIV-1 infections in Honghe Prefecture, combined with case detection characteristics, and timely detected hotspot populations and hotspot areas for HIV-1 transmission.

## Methods

### Study population

From January 2021 to December 2022, 2833 HIV-1-infected individuals were reported in Honghe Prefecture, of which 2698 were included in this study (95.2%). The inclusion criteria for subjects were: (1) age 15 years and above; (2) HIV-1 cases were reported between January 2021 and December 2022; (3) currently living in Honghe Prefecture; (4) willing to participate in the study and sign the informed consent. Participants with mental disorders, speech and hearing impairments, and other factors that may affect the completion of the survey were excluded. The Information about the subjects was collected through a questionnaire (Additional file 1). All subjects signed an informed consent form. This study was approved by the Biomedical Ethics Review Committee of the Yunnan Provincial Center for Disease Control and Prevention.

### Identification of recently infected HIV-1 cases

To identify recent HIV-1 infections among newly diagnosed cases, an HIV-1 Limiting Antigen Avidity enzyme immunoassay (LAg-Avidity EIA, Maxim Biomedical, Inc., Rockville, USA) was used to classify recent infections from long-term infections. Recent infection testing algorithms (RITAs) were used to reduce the false recent rate (FRR) of the recency assays. Prior to performing the LAg-Avidity EIA, samples with CD4^+^ T cell counts < 200 cells/µl and/or AIDS-defining illnesses were classified as long-term infections [16]. The LAg-Avidity ElA single-well preliminary screening test was first performed on all samples. Samples with a preliminary screening ODn value > 2.0 were classified as long-term infection. If the ODn value is ≤ 2.0, the samples need to be retested in a three-well assay to confirm the ODn value. In the confirmation testing, if the sample had an ODn value of 1.5 or less, the sample was classified as recent infection.

### Spatial analysis

SaTScan v10.1.2 software was used for spatial scanning statistics to detect spatial clusters of HIV-1 infection cases and recent infection cases in Honghe Prefecture in 2021–2022. To more accurately identify hot spots of HIV-1 transmission, a sensitivity analysis was conducted by setting the maximum spatial cluster size to 5%, 10%, 15%, 30% and 50% of the population at risk in the spatial window, respectively. When 50%, 30%, 15%, and 10% of the population at risk were used, the same number of spatial clusters were detected for the all-reported HIV-1 cases. For recent infections, the same number of spatial clusters were detected using 10% of the population at risk as using 30% and 15% of the population at risk. Therefore, 10% of the population at risk was chosen as the spatial size of the largest cluster. QGIS 3.28.8 was used to visualize all the data. The original map data was obtained from the National Catalogue Service for Geographic Information (http://www.webmap.cn/; accessed on May 2, 2023). Population data from the official website of the Honghe Prefectural People’s Government (http://www.hh.gov.cn/zfxxgk/fdzdgknr/tjxx_15830/202106/t20210616_527712.html; accessed on January 12, 2023).

### Statistical analysis

Statistical analysis was performed using SPSS 19.0. Logistic regression was used to calculate unadjusted odds ratios (OR) and adjusted odds ratios (AOR) and 95% confidence intervals (*CI*) for factors associated with recent HIV-1 infection. In the unadjusted analysis, the demographic and sexual characteristics, source of detection and other information were included. In the multifactor analysis, variables with a *P* < 0.1 in the unadjusted analysis were considered to control for confounding factors. *P* < 0.05 was considered statistically significant.

## Results

### Demographic characteristics of the study participants

Of the 2698 individuals eligible for inclusion, 68.2% (1840/2698) were male and 31.8% (858/2698) were female, with a male-to-female ratio of 2.14:1. The age ranged from 15 to 89 years, with those aged ≥50 years accounting for 41.1% (1108/2698). The main ethnic groups were Han (39.4%, 1063/2698), Hani (25.3%, 683/2698) and Yi (19.2%, 519/2698). Most were farmers (80.9%, 2184/2698), and 46.7% (1261/2698) were married and had a regular sexual partner. The educational level of most individuals was primary school education (46.0%, 1242/2698). More than half of the individuals (66.3%, 1790/2698) were detected through provider-initiated testing and Counselling (PITC). Heterosexual contact was the main mode of transmission (94.6%, 2551/2698) and most individuals had a history of non-marital and non-commercial heterosexual contact (53.0%, 1429/2698) (Table 1).

**Table 1:** Demographic characteristics of newly reported HIV-1 cases in Honghe Prefecture, 2021–2022

Characteristic		Total (Proportion/%)
Years of HIV diagnosis		
	2021	1717 (63.6)
	2022	981 (36.4)
Sex at birth		
	Male	1840 (68.2)
	Female	858 (31.8)
Age at HIV diagnosis (years)		
	15–34	558 (20.7)
	35–49	1032 (38.3)
	≥ 50	1108 (41.1)
Ethnic group		
	Han	1063 (39.4)
	Hani	683 (25.3)
	Yi	519 (19.2)
	Other	433 (16.0)
Occupation		
	Farmers	2184 (80.9)
	Other	514 (19.1)
Marital status		
	Unmarried	604 (22.4)
	Married	1261 (46.7)
	Divorced/Widowed	833 (30.9)
Educational level		
	No formal education	559 (20.7)
	Primary	1242 (46.0)
	Secondary	664 (24.6)
	High school and above	233 (8.6)
Screening approaches		
	PITC	1790 (66.3)
	VCT	276 (10.2)
	Testing for serodiscordant spouse or long-term sexual partner	108 (4.0)
	Other^*^	524 (19.4)
Infection routes		
	Heterosexual contact	2551 (94.6)
	MSM	84 (3.1)
	Injecting drug use	63 (2.3)
Routes of exposure		
	Non-marital non-commercial heterosexual contact	1429 (53.0)
	Commercial heterosexual sex	860 (31.9)
	HIV-positive spouse or regular sexual partner	262 (9.7)
	MSM	84 (3.1)
	Injection drug use	63 (2.3)
History of STD		
	Yes	81 (3.0)
	No	2497 (92.6)
	Unknown	120 (4.4)

Abbreviation: PITC: provider-initiated testing and counselling; VCT: voluntary counseling and testing; MSM: men who have sex with men; STD: sexually transmitted diseases

^*^Other screening approaches including community mobilization and expanded detection, COVID-19 vaccination screening, inspection of personnel in supervision places, inspection of migrant workers, medical examination of employees, and special investigation

### Characteristics of the recent infected cases

**Fig. 1:**
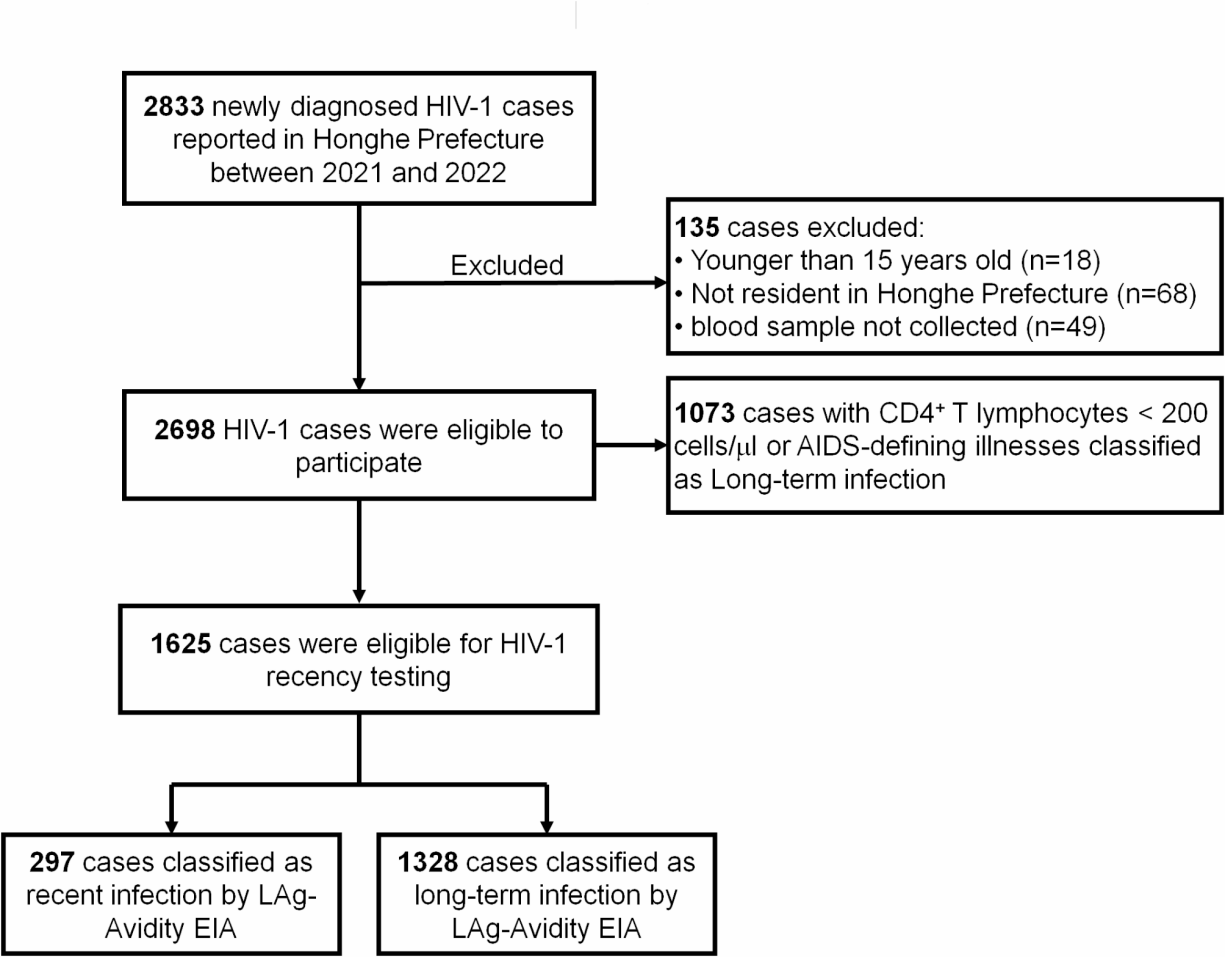
Flowchart of samples included in analyses and classified according to the recent infection testing algorithm, 2021–2022

Of the 2689 eligible cases, 1073 were classified as long-term infection based on CD4^+^ T lymphocyte count and disease status at diagnosis, and 1625 (60.2%) underwent LAg avidity enzyme immunoassay. The proportion of recent HIV-1 infection was 297 (11.0%, 95% *CI*: 9.8-12.2%). (Fig. 1).

Demographic characteristics of recent HIV-1 infection were analyzed by logistic regression (Table 2). Multivariable logistic regression analysis showed that the proportion of recent infections was higher among women (AOR = 2.61, 95% *CI*: 1.98–3.43), those aged 15–34 years (AOR = 1.71, 95% *CI*: 1.21–2.43), those with a high school education or above (AOR = 2.04, 95% *CI*: 1.24–3.37), MSM (AOR = 3.08, 95% *CI*: 1.74–5.46) and those detected through spouse/sexual partner testing (AOR = 1.96, 95% *CI*: 1.15–3.33).

**Table 2:** Demographic factors associated with recent infection among newly reported HIV-1 infections in Honghe Prefecture, 2021–2022

Factor		Total	Recent infections	%	Univariate analysis			Multivariable analysis	
					OR (95% CI)	*p*-values		AOR (95% CI)	*p*-values
Year of HIV diagnosis									
	2021	1717	186	10.8	1.00	-			
	2022	981	111	11.3	1.05 (0.82–1.35)	0.700			
Occupation									
	Farmers	2184	230	10.5	1.00	-			
	Other	514	67	13	1.27 (0.95–1.70)	0.103			
Marital status						0.191			
	Divorced/Widowed	833	78	9.4	1.00	-			
	Unmarried	604	71	11.8	1.29 (0.92–1.81)	0.143			
	Married	1261	148	11.7	1.29 (0.96–1.72)	0.087			
Ethnic group						0.007			0.020
	Han	1063	114	10.7	1.00	-		1.00	-
	Hani	683	55	8.1	0.73 (0.52–1.02)	0.066		0.74 (0.50–1.09)	0.127
	Other	433	56	12.9	1.24 (0.88–1.74)	0.223		1.22 (0.84–1.77)	0.298
	Yi	519	72	13.9	1.34 (0.98–1.84)	0.069		1.33 (0.94–1.88)	0.107
Sex at birth									
	Male	1840	157	8.5	1.00	-		1.00	-
	Female	858	140	16.3	2.09 (1.64–2.67)	< 0.001		2.61 (1.98–3.43)	< 0.001
Age at HIV diagnosis (years)						< 0.001			0.010
	35–49	1032	94	9.1	1.00	-		1.00	-
	15–34	558	88	15.8	1.87 (1.37–2.55)	< 0.001		1.71 (1.21–2.43)	0.003
	≥ 50	1108	115	10.4	1.16 (0.87–1.54)	0.323		1.06 (0.77–1.45)	0.723
Educational level						< 0.001			0.010
	No formal education	559	58	10.4	1.00	-		1.00	-
	Primary	1242	122	9.8	0.94 (0.68–1.31)	0.717		1.10 (0.77–1.58)	0.600
	Secondary	664	67	10.1	0.97 (0.67–1.41)	0.870		1.02 (0.66–1.56)	0.938
	High school and above	233	50	21.5	2.36 (1.56–3.57)	< 0.001		2.04 (1.24–3.37)	0.005
Infection routes						< 0.001			< 0.001
	Heterosexual contact	2551	267	10.5	1.00	-		1.00	-
	MSM	84	27	32.1	4.05 (2.52–6.52)	< 0.001		3.08 (1.74–5.46)	< 0.001
	Injecting drug use	63	3	4.8	0.43 (0.13–1.37)	0.154		0.43 (0.100–1.81)	0.247
Screening approaches						0.007			0.009
	PITC	1790	191	10.7	1.00	-		1.00	-
	VCT	276	38	13.8	1.34 (0.92–1.94)	0.128		1.18 (0.79–1.75)	0.419
	Testing for spouses/sexual partners of HIV-positive individuals	108	21	19.4	2.02 (1.23–3.33)	0.006		1.96 (1.15–3.33)	0.013
	Other^1^	524	47	9.0	0.83 (0.59–1.15)	0.260		0.70 (0.49–1.02)	0.065
History of STD									
	No	2497	264	10.6	1.00	-		1.00	-
	Yes	81	17	21.0	2.25 (1.30–3.89)	0.004		1.65 (0.91–2.97)	0.098
	Unknown^2^	120	16	13.3		/			

Abbreviation: PITC: provider-initiated testing and counselling; VCT: voluntary counseling and testing; STD: sexually transmitted diseases

^1^ Other screening approaches included community mobilization and expanded detection, COVID-19 vaccination screening, inspection of personnel in supervision places, inspection of migrant workers, medical examination of employees, and special investigation

^2^ Not included in logistic regression analysis

### Recent infections in the different subpopulations

Given that sex, age, route of transmission and mode of detection were associated with the proportion of recent infections, individuals were further divided into different subgroups for analysis.

First, the participants were grouped according to the transmission routes combined with sex and age (Table 3). Compared with heterosexually exposed men aged 15–34 years, heterosexually exposed men aged 35–49 years (OR = 0.48, 95% *CI*: 0.30–0.76) and ≥ 50 years (OR = 0.63, 95% *CI*: 0.41–0.99) had a lower proportion of recent infections, whereas heterosexually exposed women aged 15–34 years (OR = 1.85, 95% *CI*: 1.07–3.19) and 35–49 years (OR = 1.65, 95% *CI*: 1.05–2.58) and MSM aged 15–34 years (OR = 5.11, 95% *CI*: 2.73–9.59) had a higher proportion of recent infections.

**Table 3:** Proportion of recent HIV infections in subgroups with different routes of infection

Characteristic			Total	Recent infections	%	OR (95%*CI*)	*p*-values
Infection routes	Sex at birth	Age (years)					
Heterosexual contact	Male	15–34	356	40	11.2	1.00	-
		35–49	683	39	5.7	0.48(0.30–0.76)	0.002
		≥ 50	662	49	7.4	0.63(0.41–0.98)	0.040
	Female	15–34	132	25	18.9	1.85(1.07–3.19)	0.028
		35–49	290	50	17.2	1.65(1.05–2.58)	0.029
		≥ 50	428	64	15.0	1.39(0.91–2.12)	0.128
MSM	Male	15–34	56	22	39.3	5.11(2.72–9.59)	< 0.001
		≥ 35	28	5	17.9	1.72(0.62–4.77)	0.299
Injecting drug use	Male	≥ 15	55	2	3.6	0.30(0.07–1.27)	0.102
	Female	≥ 15	8	1	12.5	1.13(0.14–9.41)	0.911

Abbreviation: MSM: men who have sex with men

Second, the participants were grouped according to the mode of exposure combined with sex (Table 4). Compared with men infected through non-marital non–commercial heterosexual contact, MSM (OR = 5.59, 95% *CI*: 3.30–9.47), women infected through women infected through non-marital non-commercial heterosexual contact (OR = 2.10, 95%*CI*: 1.50–2.95) and positive spousal exposure (OR = 3.03, 95% *CI*: 1.96–4.69) had a higher proportion of recent infections.

**Table 4:** Proportion of recent HIV infections in subgroups with different routes of exposure

Characteristic		Total	Recent infections	%	OR (95%CI)	*p*-values
Sex at birth	Routes of exposure					
Male	Non-marital non-commercial heterosexual contact	794	62	7.8	1.00	-
	Commercial heterosexual contact	836	61	7.3	0.93(0.64–1.34)	0.696
	Exposure of HIV-positive spouses/regular sexual partners	71	5	7.0	0.89(0.35–2.30)	0.817
	MSM	84	27	32.1	5.59(3.30–9.47)	< 0.001
	Injecting drug use	55	2	3.6	0.45(0.11–1.87)	0.270
Female	Non-marital non-commercial heterosexual contact	635	96	15.1	2.10(1.50–2.95)	< 0.001
	Commercial heterosexual contact	24	4	16.7	2.36(0.78–7.12)	0.127
	Exposure of HIV-positive spouses/regular sexual partners	191	39	20.4	3.03(1.96–4.69)	< 0.001
	Injecting drug use	8	1	12.5	1.69(0.20-13.93)	0.627

Abbreviation: MSM: men who have sex with men

Thirdly, the participants were grouped according to the screening approaches combined with sex (Table 5). The proportion of recent infections among women screened through PITC (OR = 2.03, 95% *CI*: 1.50–2.76) and spouse/fixed partner testing (OR = 4.10, 95% *CI*: 2.19–7.67) were higher.

**Table 5:** Proportion of recent HIV infections in subgroups with different sources of detection

Characteristic		Total	Recent infections	%	OR (95%CI)	*p*-values
Sex at birth	Screening approaches					
Male	PITC	1229	103	8.4	1.00	-
	VCT	193	24	12.4	1.55(0.97–2.49)	0.068
	Spouses/sexual partners Testing	53	6	11.3	1.40(0.58–3.34)	0.454
	Other^1^	365	24	6.6	0.77(0.49–1.22)	0.265
Female	PITC	561	88	15.7	2.03(1.50–2.76)	< 0.001
	VCT	83	14	16.9	2.22(1.21–4.08)	0.010
	Spouses/sexual partners Testing	55	15	27.3	4.10(2.19–7.67)	< 0.001
	Other^1^	159	23	14.5	1.85(1.14–3.01)	0.013

Abbreviation: PITC: provider-initiated testing and counselling; VCT: voluntary counseling and testing

^1^ Other screening approaches including community mobilization and expanded detection, COVID-19 vaccination screening, inspection of personnel in supervision places, inspection of migrant workers, medical examination of employees, and special investigation

### Spatial analysis for HIV-1 recent infections

**Fig. 2:**
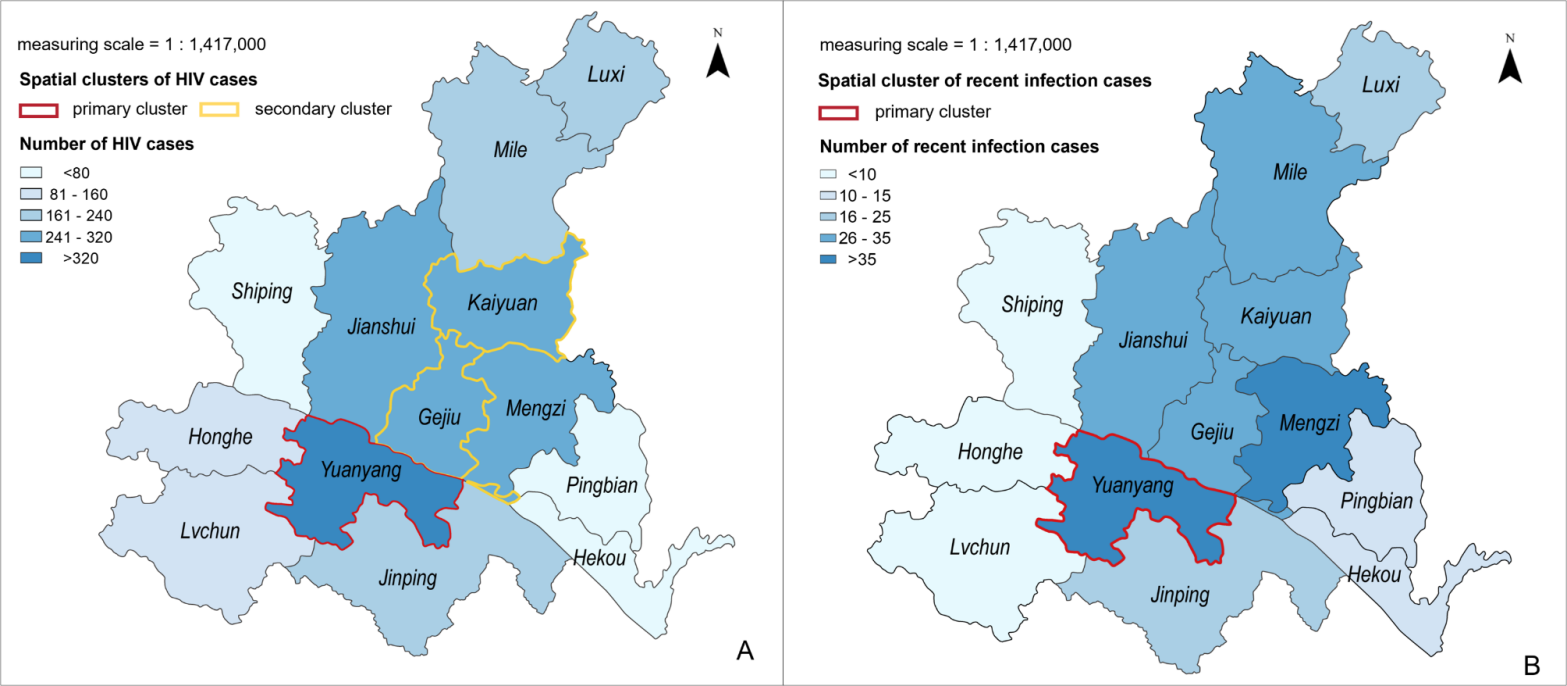
Spatial cluster of recent HIV infection cases in Honghe Prefecture, 2021–2022. **a** The geographic distribution and spatial cluster of newly reported HIV-1 cases in Honghe Prefecture, 2021–2022. **b** The geographic distribution and spatial cluster of recent infected cases in Honghe Prefecture, 2021–2022. The map content approval number: YunS(2024)15

The choropleth maps showed that the overall HIV-1 infections and recent HIV-1 infections were mainly distributed in the central and southeastern parts of Honghe Prefecture (Fig. 2). Spatial scan analysis revealed three clusters of newly reported HIV-1 cases (all *P* < 0.001), with the primary cluster located in Yuanyang (*RR* = 2.97) and the secondary cluster located in Kaiyuan (*RR* = 1.50) and Gejiu (*RR* = 1.25) (Fig. 2A). A primary cluster of recent infections was detected in Yuanyang (*P* < 0.001, *RR* = 2.05) (Fig. 2B).

## Discussion

In this study, a survey of recent HIV-1 infection was conducted among newly diagnosed cases in Honghe Prefecture. Together with the demographic and spatial distribution characteristics, the potential factors associated with recent HIV-1 infection were further analyzed. Timely tracking of recent HIV-1 transmission is key to promoting early detection and effectively controlling the risk of transmission.

The proportion of recent infections among newly diagnosed HIV-1 cases in Honghe Prefecture in 2021–2022 was 11.0%, which is higher than the that in the Yunnan Province in 2015 (9.3%, χ^2^ = 6.05, *P* = 0.014) [14]. Since 2016, to promote the 90-90-90 targets, Yunnan Province has implemented HIV expanded testing strategies, and the testing coverage increased from 25.2 to 74.8% during 2015–2020 [17]. As a key area for AIDS prevention and control in Yunnan Province, Honghe Prefecture has responded positively the HIV expanded testing strategies. In addition to routine VCT and PITC, the expanded HIV testing also included HIV mobilization testing in community and special settings and HIV screening integrated with medical examination, labor export programs and COVID-19 vaccination. These measures might have contributed to the early detection of infected persons, potentially increasing the proportion of recent infections. However, the proportion of recent infections remains relatively low, such as 14.2% in Ethiopia and 20.5% in Korea [18, 19]. Inadequate HIV detection and delayed diagnosis are major challenges facing the HIV response [15]. To increase the detection rate of new HIV infections, innovative testing models, such as the promotion of Internet + facilitated testing and the implementation of HIV partner tracing and testing, are needed in addition to routine measures.

As found in the previous study, the proportion of recent HIV-1 infections was higher among women than among men [8, 20]. The analysis of subpopulations further revealed that the proportion of recent infections among heterosexually contact exposed women was higher than that among heterosexually contact exposed men in the different age groups. Due to physiological and sociological factors, women are at higher risk of HIV-1 infection than men [21, 22]. In 2022, approximately 210,000 young women (15–24 years) worldwide were infected with HIV, which was much higher than the number of infected young men (15–24 years) (140,000) [23]. The observed higher proportion of recent infections among women might be partially attributed to their better access to HIV testing services [24]. Women could be tested for HIV as part of basic public health services, such as women’s health care, maternity care, and so on. These measures could improve access to HIV testing for women and maximize the identification and management of female sources of HIV infection.

Our results also showed that the higher proportion of recent infections was among young people, especially in the 15–34 age group. Among heterosexually and homosexually exposed men, the 15–34 age group had the highest proportion of recent infections. Among heterosexually exposed women, the 15–34 age group had a higher proportion of recent infections than the other age group. At the national level in China, the HIV epidemic is rising rapidly among young people aged 15–24 [25]. Therefore, young people are one of the populations that need to be targeted for HIV/AIDS prevention and control. Further targeted interventions are needed for this subpopulation, including effectively improving their awareness of HIV/AIDS prevention. In addition, the overall proportion of recent infections, as well as the proportion of recent infections in different subpopulations, showed a decreasing trend with age increasing. This might be related to older adults’ lack of risk awareness and lower awareness of active detection after high-risk sexual behavior [26]. In this study, 71.3% (790/1108) of individuals aged ≥50 years were detected through PITC. As most older people had underlying health conditions, symptoms of HIV infection were often masked. The long interval between infection and case detection may result in missing opportunities for early testing.

Furthermore, our results suggested that recent HIV-1 infection was associated with educational status, the proportion of recent infections increased along with increasing educational status. As found, individuals with a high school education or higher were twice as likely to be found as recent infections than those who were no formal education (AOR = 2.04, 95% CI: 1.24–3.37). Based on the associated studies, we speculated that the lower proportion of recent infections among individuals with low education may be related to their lack of awareness of HIV testing, while the high proportion of recent infections among individuals highly educated may be related to their greater awareness of HIV testing and high level of knowledge about HIV/AIDS prevention and control [27, 28]. These differences indicate a need to consider strengthening AIDS promotion and education tailored to people with different levels of education. For people with low levels of education, the priority should be to reduce high-risk behaviors and increase the awareness of active HIV testing; for people with high levels of education, the priority should be to improve the effectiveness and accessibility of intervention services.

In this study, the proportion of recent infections in MSM (32.1%) was much higher than in heterosexual transmission (10.5%). The previous studies showed that MSM had a higher rate of unprotected sex, multiple sexual partners and recreational drug use [29–31]. Meanwhile, the rate of condom use during sexual intercourse among MSM was less than 60% [32, 33]. The relevant studies also showed that the HIV incidence among MSM was higher than heterosexual transmission [34, 35]. However, more than 50% of MSM had regular HIV testing [31, 36]. MSM constitute a particular group and may hesitate to reveal their identity given the cultural context of China. Interventions for this group mainly rely on community-based organizations (CBOs) and are carried out through peer-driven approaches, including regular follow-up testing for MSM participating in intervention programs [37]. Furthermore, new methods utilizing mobile phones and internet platforms have been introduced, which can help to some extent in the early identification and referral of infected individuals for treatment [38]. To reduce new infections among MSM, the coverage and effectiveness of MSM interventions through CBOs should be increased, including improving awareness of condom use and promoting pre-exposure prophylaxis (PrEP) and post-exposure prophylaxis (PEP) services.

Although there was no statistical difference, the proportion of recent infections among IDUs was lower than that among the heterosexual population. Effective interventions, such as needle exchange and methadone substitution therapy, were implemented for IDUs, which may have contributed to a decrease in HIV-1 infections among this group [39]. In recent years, the proportion of IDUs among newly reported cases in Yunnan Province has remained low [40]. Nevertheless, due to the illegal nature of injecting drug use, some IDUs may be reluctant to access intervention services and may not be easily reached by testing services.

PITC relies on the established medical service system to provide HIV testing services under the condition of informed consent. PITC is currently the primary means of identifying infected individuals, and this has been recognized in Yunnan Province’s prevention and control strategies. Consistent with the overall situation in Yunnan Province, PITC was the predominant route for HIV case detection in Honghe Prefecture. In our studies, the proportion of recent infections among cases detected through PITC was 10.7%, which was close to the average proportion of recent infections (11.0%). Unlike PITC, VCT is a way to actively seek HIV testing after high-risk behavior. In theory, VCT should have a higher rate of detection of recent infections. In this study, the proportion of recent infections detected by VCT (13.8%) was slightly higher than PITC, but there was no statistically significant difference between the two groups. This suggested, to some extent, that the general population had low awareness of actively seeking HIV testing after engaging in high-risk behaviors or was unaware of available HIV testing services. From an individual health perspective, the knowledge of HIV testing and awareness of proactive testing should be further strengthened. From a service delivery perspective, the advantage and effectiveness of VCTs should be strengthened to avoid homogenization of VCTs and PITCs.

The highest proportion of recent infections (19.4%) was found among individuals detected through spouse/sexual partner testing, suggesting that spouses or sexual partners of HIV-1-positive individuals were vulnerable. In particular, the proportion of recent infections among women detected in this way (27.3%) was higher than that among men (11.3%), suggesting that the female partner was more likely to be infected in HIV serodiscordant couples. Therefore, to reduce the risk of HIV transmission and prevent positive conversion among HIV serodiscordant couples, it is necessary to increase spouse/partner notification, implement condom distribution and use, and standardize antiviral treatment for the positive side.

In the implementation of the expanded HIV testing strategy, measures for HIV testing were widely explored and practiced, such as community mobilization testing, testing for migrant workers, testing combined with basic public health services and so on. The proportion of recent infections detected by these detection methods (9.0%) was slightly lower than by PITC, but the difference was not statistically significant. These detection methods expanded the coverage of detection in practice. To effectively increase the discovery rate, it is necessary to further explore the relevance of detection, refine detection plans, and create an appropriate policy support environment.

Spatial clusters and “hotspots” of disease indicate an increased risk of disease. Hotspots can provide clues to disease etiology and risk factors [41]. Using spatial scan statistics, we simultaneously detected significant clusters of HIV-1 infection cases and recent HIV-1 infections in Yuanyang, indicating that Yuanyang had a high risk of HIV-1 transmission. In Kaiyuan and Gejiu, we found a cluster of HIV-1 infection cases, but no cluster of recent HIV-1 infections. This suggested that there could be some delay in diagnosing people infected with HIV-1 in these areas. More attention should be paid to these areas to avoid increasing the risk of epidemic spread due to delayed diagnosis in the future. Spatial analysis of recent HIV-1 infections can reveal the epidemic trend of the current epidemic and identify potential transmission hotspots. For policy makers and planners, this information can guide the formulation of effective public health intervention strategies in hotspot areas, inform epidemic prevention and control, and provide data to support resource configuration.

There are also limitations to this study. First, the cross-sectional design precludes causal inferences between demographic factors and recent infections. Second, despite the use of a recent infection algorithm, it cannot be completely excluded that a small number of long-term infections were misclassified as recent infections due to the limitations of the detection method. Third, due to the short time span of the study, it is not possible to reflect the trend of recent infections.

## Conclusion

In this study, based on HIV case-report surveillance, a survey of recent HIV-1 infection was conducted in one of the most HIV-1-affected areas in Yunnan Province, China. The overall proportion of recent infections was relatively low in the local area, suggesting that HIV diagnosis is still delayed. More proactive interventions are needed to improve early diagnosis and hence early initiation of ART. The demographic and behavioral characteristics, such as sex, age, education, exposure mode, were associated with the detection of recent infection. Further analysis revealed the different key subpopulations. Spatial analysis of recent HIV-1 infections showed that there were differences in the distribution of recent HIV-1 infections, with a cluster of recent infections detected in Yuanyang. This study informed the use of recency testing for surveillance. The results provided a scientific reference to guide the adjustment of AIDS prevention and control strategies.

## Electronic supplementary material

Below is the link to the electronic supplementary material.

**Supplementary Material 1**: **Additional file 1**: Risk factor questionnaire for newly reported HIV/AIDS cases.

## Data Availability

This paper is based on public health surveillance data collected continuously by the Honghe Centers for Disease Control and Prevention in Honghe Prefecture. The study database used and/or analysed in the current study is available from the corresponding author upon reasonable request.

## Abbreviations

AHI: Acute HIV infection
AIDS: Acquired immune deficiency syndrome
AOR: Adjusted odds ratio
ART: Antiretroviral therapy
CI: Confidence interval
EHI: Early HIV infection
FRR: False recent rate
IDUs: Injecting drug users
LLR: Log-likelihood ratio
LT: Long-term infection
MSM: Men having sex with men
OR: Odds ratio
PEP: Post-exposure prophylaxis
PITC: Provider-initiated testing and counseling
PrEP: Pre-exposure prophylaxis
RHI: Recent HIV infection
RITA: Recent infection testing algorithm
RR: Relative risk
STD: Sexually transmitted disease
VCT: Voluntary counseling and testing
WHO: World health organization
