# Supplementary material for: Analysis of HIV-1 recent infections and hotspot areas in a bordering area in Yunnan Province, China
Source: BMC Public Health. 2025 Mar 24;25:1122. doi: 10.1186/s12889-025-21877-3 (PMC11934778; doi:10.1186/s12889-025-21877-3)
Supplement: Supplementary file 1 — Supplementary Material 1: Additional file 1: Risk factor questionnaire for newly reported HIV/AIDS cases. [file 12889_2025_21877_MOESM1_ESM.docx]

***Additional file 1***

**Investigation ID： Case number:**

**Risk factor questionnaire for newly reported HIV/AIDS cases**

We have invited you here because we want to get a better understanding of your HIV status, which will help us with your future treatment and also help us to improve the AIDS prevention and treatment in the local area. We hope that you will support us in our work.

**1. General demographic information**

1.1 Sex at birth:

(1) Male

(2) Female

1.2 Your date of birth: DD/MM/YYYY

1.3 Your ethnicity:

(1) Han

(2) Ethnic minority (please specify)

1.4 Your marital status:

(1) Unmarried

(2) Married

(3) Divorced/Widowed

1.5 Your place of household registration：

(1) County where case was reported

(2) Other county of Honghe Prefecture (please specify)

(3) Other prefecture in Yunnan Province (please specify)

(4) Other provinces (please specify)

1.6 Your current place of residence

(1) County where case was reported

(2) Other county of Honghe Prefecture (please specify)

(3) Other prefecture in Yunnan Province (please specify)

(4) Other provinces (please specify)

1.7 Your level of education：

(1) Illiterate

(2) Primary

(3) Secondary

(4) High school or technical secondary school

(5) College degree or above

1.8 Your Occupation:

(1) Student

(2) Worker

(3) Farmer

(4) Cadres and Employee

(5) Retired Personnel

(6) Housework and unemployed

(7) Sole proprietorship

(8) Business service

(9) Others (please specify)

**2. Behavioral information**

**Exposure routes:**

(1) Positive spouse

(2) Positive regular sexual partner

(3) Non-marital and non-commercial heterosexual sex

(4) Commercial heterosexual sex

(5) Male sex

(6) Injecting drug use

(7) Maternal positive

(8) Others

**3. Sexually transmitted diseases**

3.1 Have you ever been diagnosed with an STD：

(1) Yes

(2) No (skip to 4)

3.2 What kind of sexually transmitted diseases have you been diagnosed with (you can choose more than one):

(1) Gonorrhea

(2) Syphilis

(3) Genital tract chlamydia trachomatis infection

(4) Genital warts

(5) Genital herpes

(6) Other (please specify) __________

**4. What screening approaches did you use to know you were infected with HIV?**

(1) Provider-initiated testing and counselling (In routine medical, preventive and physical examination services, medical personnel should take the initiative to disseminate information about AIDS and provide AIDS testing services)

(2) Voluntary counseling and testing

(3) Testing for spouses or sexual partners of HIV-positive individuals

(4) Other (community mobilization and expanded detection, COVID-19 vaccination screening, testing of staff in supervisory positions, testing of migrant workers, medical examination of employees and special investigations)

**Investigator's signature: ____________**

**Quality controller's signature: _________**

**Date of the investigation：**
